# Association between changes in body composition and progression of liver fibrosis in patients with type 2 diabetes mellitus

**DOI:** 10.3389/fnut.2024.1476467

**Published:** 2024-10-21

**Authors:** Yuxi Lin, Zhixing Liang, Xiaofang Liu, Yutian Chong

**Affiliations:** ^1^Department of Infectious Diseases, The Third Affiliated Hospital of Sun Yat-sen University, Guangzhou, China; ^2^Guangdong Provincial Key Laboratory of Liver Disease Research, The Third Affiliated Hospital of Sun Yat-sen University, Guangzhou, China; ^3^Department of Hepatic Surgery and Liver Transplantation Center, The Third Affiliated Hospital of Sun Yat-sen University, Guangzhou, China; ^4^Department of Neurology, The Third Affiliated Hospital of Sun Yat-sen University, Guangzhou, China

**Keywords:** body composition, body mass index, liver fibrosis progression, type 2 diabetes mellitus, muscle fat ratio

## Abstract

**Aim:**

The correlation between type 2 diabetes mellitus (T2DM) and the occurrence of liver fibrosis is well-established. However, the longitudinal association between body composition and liver fibrosis progression in patients with T2DM remains incompletely explored.

**Methods:**

Total of 390 patients with T2DM underwent body composition assessments, followed by a median duration of 2.13 years. The calculated parameters included body mass index (BMI), fat mass index (FMI), trunk fat mass index (TFMI), appendicular skeletal muscle mass index (ASMI), muscle/fat mass ratio (M/F) and appendicular skeletal muscle mass/trunk fat mass ratio (A/T). Liver fibrosis was evaluated through liver stiffness measurement (LSM). Patients were classified according to BMI and body composition, followed by a comprehensive investigation into the impact of body composition changes on liver fibrosis outcomes.

**Results:**

Among 72 patients with incident advanced liver fibrosis at readmission, ΔBMI, ΔFMI and ΔTFMI increased, while ΔM/F and ΔA/T decreased. Individuals who kept obese had a dramatically elevated hazard of incident advanced liver fibrosis compared to those who kept non-obese, with an adjusted odds ratio of 3.464. When TFMI heightened, the hazard of incident advanced liver fibrosis was 3.601 times higher compared to the decreased group. Additionally, individuals in increased ASMI and A/T groups showed a slight advantage in preventing incident advanced liver fibrosis compared to the stable groups.

**Conclusion:**

Stable obesity was associated with a greater hazard of liver fibrosis advancement, and an increase in TFMI may promote the progression of liver fibrosis. Maintaining a balanced muscle/fat ratio appeared to help prevent the progression.

## 1 Introduction

Researchers have emphasized the significance of liver fibrosis severity as a pivotal determinant of long-term prognosis, exhibiting strong correlations with both hepatic and extra-hepatic events as well as mortality (1, 2). Relevant studies have previously established a robust correlation between type 2 diabetes mellitus (T2DM) and the initiation as well as progression of liver fibrosis (3, 4). The underlying pathological mechanism suggests that elevated blood glucose levels play a direct role in inducing hepatotoxicity, leading to hepatocellular injury and eventual mortality. Therefore, the assessment of liver fibrosis progression in individuals with T2DM holds significant importance. Although the assessment of liver fibrosis staging relies on liver biopsy as the gold standard (5), its limitations encompass exorbitant expenses, invasiveness, and suboptimal adherence. Consequently, the recent recommendation is to employ non-invasive methodologies such as ultrasound transient elastography (TE) (6).

The weight change serves as an indicator of an individual’s lifelong trajectory toward optimal health (7, 8). The weight fluctuations observed in individuals with T2DM are influenced by multiple factors. Relevant investigations have indicated that the underlying mechanisms contributing to the adverse effects of weight fluctuations across different life stages may exhibit variations (7, 9). For instance, initial weight gain primarily arises from lipid accumulation (10–12), while it is frequently attributed to a decline in muscle mass over time (13, 14). Importantly, even when body weight remains stable, the distribution of adipose tissue and muscle mass can vary significantly. Notably, recent research has established a significant association between sarcopenia and non-alcoholic fatty liver disease (NAFLD), which is one of the major risks of liver fibrosis (15). The association between the two has been further substantiated by another study, independent of obesity and insulin resistance (16). The study findings also indicated that an increased risk of liver fibrosis progression was associated with both weight gain and obesity (17).

When assessing T2DM patients during subsequent visits, it is crucial to acknowledge that changes in weight may not serve as the exclusive indicator of liver fibrosis. In spite of numerous researches conducted on the correlation between fluctuations in body weight and the development of liver fibrosis among adults (18–22), the impact of changes in body composition on the prognosis of liver fibrosis in patients with T2DM remains unknown. Furthermore, the majority of researches have utilized cross-sectional methodologies, and a longitudinal cohort study that is pertinent to this topic remains absent. We hoped to compare the frequency of incident advanced liver fibrosis and non-advanced liver fibrosis in patients with T2DM at baseline and readmission, and further explore potential body composition parameters that may contribute to preventing advanced liver fibrosis progression.

## 2 Materials and methods

### 2.1 Study population

**Figure 1:**
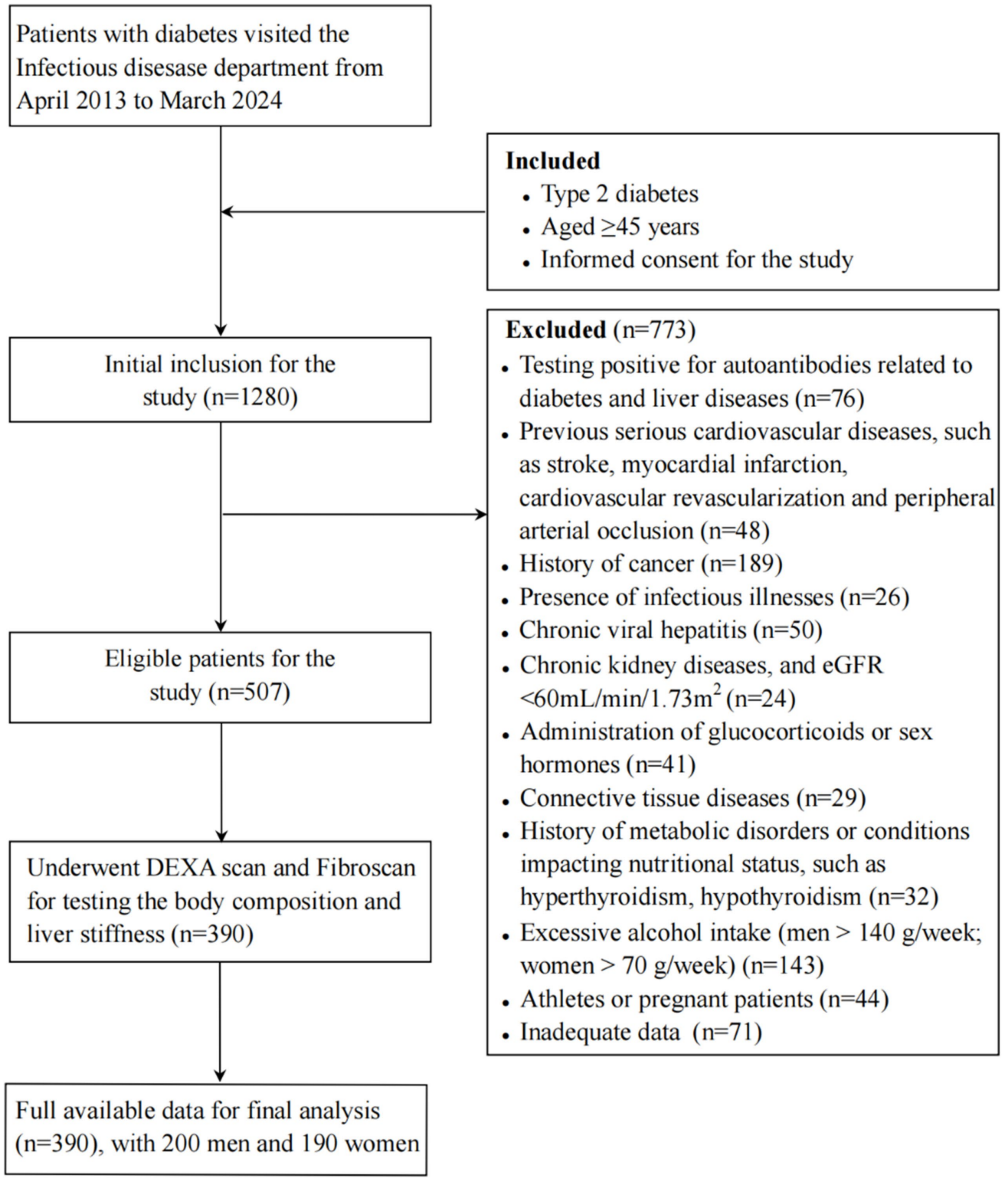
Study flowchart Note: eGFR, estimated glomerular filtration rate; DEXA, dual-energy X-ray absorptiometry.

This was a retrospective cohort study conducted in the Department of Infectious Diseases, the Third Affiliated Hospital of Sun Yat-sen University. We systematically selected 1,280 participants by recruiting every third hospitalized patient from April 1, 2013, to March 30, 2024. After the preliminary assessment, 507 individuals participated in subsequent phase of the study. Those lacking comprehensive data were omitted from the examination. Ultimately, the sample size was narrowed down to 390 participants, comprising 200 males and 190 females. The sample size achieved sufficient power to detect the expected differences with the given effect size. The median follow-up duration was 2.13 years. The study flowchart is displayed in Figure 1.

Inclusion criteria: (1) Aged ≥45 years who satisfied the 2021 American Diabetes Association diagnostic standards for T2DM and were experiencing antidiabetic drug treatment (23); (2) Had complete data on body composition and liver stiffness assessment; (3) Understood the study’s purpose and voluntary participation.

Exclusion criteria: (1) Declined participation; (2) Had other types of diabetes; (3) Critically ill patients who had ketoacidosis, hyperosmotic nonketotic coma, cirrhosis, chronic viral hepatitis (including hepatitis B and C virus infection), infectious illnesses, malignant tumors or autoimmune disease, etc.; (4) Had muscle loss due to poisoning, drug abuse or anti-inflammatory or hormone drugs uses; (5) History of severe cardiovascular diseases; (6) History of metabolic disorders affecting nutritional status; (7) Excessive alcohol intake (men >140 g/week; women >70 g/week) (24); (8) Tested positive for autoantibodies associated with diabetes and hepatic disorders; (9) Athletes or pregnant women.

### 2.2 Data collection

Experienced physicians obtained comprehensive clinical data from all participants, including gender, age, disease course, and family history, etc. To guarantee the precision and legitimacy of the data, patient’s identification number and admission number were securely obtained, and medical records were reviewed. Measured the individual’s weight and height in the morning (model: RGZ-120-RT). After a 15-min rest, blood pressure and body mass index (BMI) was measured. BMI = weight (kg) / height^2^ (m^2^). The waist to hip ratio (WHR) = waist circumference (cm) / hip circumference (cm). BMI ≥28 was Obesity (25).

The venous blood samples were collected following a 10-h fasting. The concentrations of total cholesterol (TC), triglyceride (TG), high-density lipoprotein cholesterol (HDL-c), low-density lipoprotein cholesterol (LDL-c), aspartate aminotransferase (AST), alanine aminotransferase (ALT), *γ*-glutamyl transferase (GGT) and creatinine (Cr) were measured using Siemens ADVIA 2400 automatic biochemical analyzer. Additionally, C-reactive protein (CRP) levels, platelet counts (PLT), international normalized ratio (INR) values for prothrombin time, albumin (ALB) levels and fasting plasma glucose (FPG) concentrations were determined. The estimated glomerular filtration rate (eGFR) = 186× (serum Cr [μmol/L]/88.41)–1.154 × age–0.203 (×0.742 female) (12). High-performance liquid chromatography (VARIANTII; Bio-Rad, CA, United States) was used to determine glycosylated hemoglobin (HbA1c) levels.

### 2.3 Liver fibrosis assessment

Controlled attenuation parameter (CAP) and liver stiffness measurement (LSM) were obtained for each patient using available TE evaluation (FibroScan; Echosens^®TM^, Echosens, Paris, France). The intra- and inter-assay coefficients of variation for FibroScan were 0.78 and 0.83%, respectively. LSM scores were evaluated for the detection of liver fibrosis. To estimate reliability, we computed the ratio of the LSM interquartile range (IQR) to its median. LSM must be at least 10 kPa with a success rate of at least 60%, and a ratio of IQR to median LSM should not be exceed 30%. The validity of CAP is only confirmed when the corresponding LSM meets these criteria.

LSM < 8.2 kPa was defined as F0. The presence of significant fibrosis (≥F1) was indicated by a median LSM value of ≥8.2 kPa, while advanced fibrosis (F2) and cirrhosis (F3) were indicated by LSM values of ≥9.7 kPa and ≥ 13.6 kPa, respectively (6). Newly occurring F2 and F3 grades of liver fibrosis at readmission was referred to as incident advanced liver fibrosis (incident F2-3). A transition from F2 to either F0 or F1 was defined as incident non-advanced liver fibrosis (incident F0-1). Patients with cirrhosis at baseline have been excluded from this study.

### 2.4 Body composition examination

Dual-energy X-ray absorptiometry (DEXA, American GELUNAR Company, Prodigy Type) was utilized to assess body composition. The whole-body fat mass index (FMI) = whole-body fat mass (kg)/height^2^ (m^2^); whole-body muscle mass index (MMI) = muscle mass (kg)/height^2^ (m^2^); trunk fat mass index (TFMI) = trunk fat mass (kg)/height^2^ (m^2^); appendicular skeletal muscle mass index (ASMI) = appendicular skeletal muscle mass (kg)/height^2^ (m^2^). M/F = whole-body muscle mass (kg)/whole-body fat mass (kg); A/T = appendicular skeletal muscle mass (kg)/trunk fat mass (kg). The change value represented the disparity between measurements at baseline and readmission. The adjustment of annual change rates based on the duration of follow-up in years. The intra- and inter-assay coefficients of variation for DEXA were 0.64 and 0.80%, respectively.

### 2.5 Grouping criteria

We assessed BMI and body composition indexes at baseline and readmission. The patterns of BMI change were categorized into four groups: stable non-obese (<28 kg/m^2^), weight losing (baseline ≥28 kg/m^2^ and readmission <28 kg/m^2^), weight gaining (baseline <28 kg/m^2^ and readmission ≥28 kg/m^2^), and stable obese (≥28 kg/m^2^) (8).

Changes in body composition were quantified by the differences between baseline and readmission measurement values of BMI (ΔBMI), FMI (ΔFMI), MMI (ΔMMI), M/F (ΔM/F), TFMI (ΔTFMI), ASMI (ΔASMI) and A/T (ΔA/T). A previous investigation revealed that patients in the intervention group exhibited a significant increase of approximately 3% in leg muscle mass compared to those without any special intervention. Therefore, we established 3% as the threshold value. The body composition indexes were categorized as decreasing, stabilizing, and increasing according to ΔFMI, ΔMMI, ΔM/F, ΔTFMI, ΔASMI, and ΔA/T < −3%, −3 to 3, and > 3%, respectively (26).

### 2.6 Statistical analysis

SPSS for Windows (version 25.0) was utilized for statistical analysis, and *p* < 0.05 indicated significance. We specified the primary outcomes of interest for our study and established the desired significance level (*α* = 0.05) and power (1-*β* = 0.80), which is commonly accepted in clinical research. Suitable sampling weight analysis was added in analysis. When the information is not collected, information is lost after being collected, and the information is collected, identified as incorrect, and deleted, the data was identified as missing. Inserted techniques such as multiple imputations were chosen to minimize bias and maintain the integrity of the dataset. Continuous variables were presented as means with standard deviations (SDs) or medians with IQRs, evaluating group differences using an independent sample t-test or non-parametric test. Categorical variables were presented as frequencies and percentages, evaluating group differences using χ^2^ test. Pearson’s correlation coefficient was employed to assess univariate association between body composition and LSM. Bivariate logistic regression was conducted to investigate the association between weight change patterns and the occurrence of advanced liver fibrosis, with odds ratios (ORs) and 95% confidence intervals (CIs) being reported. Binary logistic regression analyzed the correlations between ΔFMI, ΔMMI, ΔM/F, ΔTFMI, ΔASMI, ΔA/T and incident advanced liver fibrosis, with adjusted findings presented as OR and 95% CI.

## 3 Results

### 3.1 Patient characteristics

The median duration of follow-up for the 390 readmitted individuals (200 men and 190 women) was 2.13 years, and with an average age of 61.02 ± 12.05 years. Patients with advanced liver fibrosis and non-advanced liver fibrosis at baseline were separately compared (Table 1). Statistically significant differences were observed in LSM, CAP, duration, DBP, TC, TG, ALT, AST, ALB levels and the prevalence of obesity among patients at baseline and readmission. At readmission, patients in advanced liver fibrosis group demonstrated a decrease in FMI and TFMI, while the M/F and A/T increased (*p* < 0.05; Supplementary Table S1).

**Table 1:** Comparison of characteristics between baseline and readmitted subjects.

Variables	Advanced liver fibrosis at baseline(*n* = 129)	Readmitted patients(*n* = 129)	*p*	Non-advanced liver fibrosis at baseline(*n* = 261)	Readmitted patients(*n* = 261)	*p*
Duration (years)	6.97 ± 6.36	8.56 ± 6.95	**<0.001**	8.65 ± 6.88	9.49 ± 7.21	**0.014**
Male (n, %)	66(51.2)	/	/	134(51.3)	/	/
BMI (kg/m^2^)	25.83 ± 3.20	26.31 ± 8.77	0.053	22.86 ± 3.03	23.01 ± 3.52	0.078
WHR	84.32 ± 9.95	91.62 ± 8.97	**<0.001**	82.07 ± 9.34	89.70 ± 10.43	**<0.001**
SBP (mmHg)	137.14 ± 19.30	135.04 ± 20.43	**0.026**	138.61 ± 20.05	138.55 ± 21.59	0.952
DBP (mmHg)	81.42 ± 11.29	77.81 ± 10.95	**<0.001**	77.98 ± 10.45	75.94 ± 10.90	**<0.001**
Obesity (n, %)	41(31.8)	46(35.7)	**0.012**	23(8.8)	27(10.3)	**0.023**
HT (n, %)	12(9.3)	17(13.2)	0.179	31(11.9)	33(12.6)	0.129
Current smoking (n, %)	48(37.2)	51(39.5)	0.541	25(9.6)	29(11.1)	0.421
Alcoholic consumption (n, %)	56(43.4)	43(33.3)	0.365	37(14.2)	34(13.0)	0.190
Antidiabetic treatments						
Drug naive, n (%)	34(26.4)	33(25.6)	0.122	66(25.3)	45(17.2)	**0.021**
Insulin, n (%)	40(31.0)	45(34.9)	0.239	87(33.3)	72(27.6)	**0.018**
Secretagogues, n (%)	23(17.8)	21(16.3)	0.340	45(17.2)	43(16.5)	0.098
Metformin, n (%)	49(38.0)	60(46.5)	**0.005**	65(24.9)	50(19.2)	**0.010**
TZDs, n (%)	33(25.6)	45(34.9)	**0.013**	38(14.6)	41(15.7)	0.078
AGIs, n (%)	41(31.8)	30(23.3)	**0.041**	42(16.1)	39(14.9)	0.061
DPP-4Is, n (%)	32(24.8)	28(21.7)	0.088	44(16.9)	40(15.3)	0.054
SGLT-2Is, n (%)	28(21.7)	34(26.4)	0.120	54(20.7)	59(22.6)	0.103
GLP-1RAs, n (%)	20(15.5)	24(18.6)	0.051	50(19.2)	52(19.9)	0.198
Statin use, n (%)	67(51.9)	51(39.5)	**0.039**	88(33.7)	96(36.8)	0.135
Biochemical data						
TC (mmol/L)	4.92 ± 1.29	4.73 ± 1.23	**<0.001**	4.68 ± 1.44	4.34 ± 1.20	**0.003**
TG (mmol/L)	2.25 ± 2.15	1.30 ± 0.82	**<0.001**	2.22 ± 1.89	1.49 ± 1.05	**<0.001**
HDL-c (mmol/L)	1.28 ± 0.41	1.14 ± 0.31	**<0.001**	1.01 ± 0.27	1.13 ± 0.38	**<0.001**
LDL-c (mmol/L)	2.99 ± 1.10	2.90 ± 1.02	0.085	2.73 ± 1.01	2.84 ± 1.00	**0.021**
ALT (U/L)	25.00(18.00–38.25)	20.00(14.00–31.00)	**<0.001**	21.00(15.00–31.00)	17.00(12.00–24.00)	**<0.001**
AST (U/L)	21.00(17.00–28.00)	20.00(16.00–27.00)	**<0.001**	21.00(17.00–26.00)	19.00(15.00–25.00)	**<0.001**
GGT (U/L)	32.00(22.00–54.00)	24.00(17.00–40.00)	**<0.001**	27.00(18.00–44.00)	20.00(14.00–35.00)	**<0.001**
Cr (umol/L)	76.26 ± 37.82	74.73 ± 32.96	0.287	67.18 ± 31.45	66.04 ± 55.27	0.174
eGFR (ml/min/1.73m^2^)	97.50 ± 20.88	96.26 ± 25.23	0.743	92.26 ± 25.66	86.05 ± 33.61	0.211
PLT (10^9^/L)	226.33 ± 71.85	223.17 ± 82.01	0.393	250.28 ± 72.37	246.81 ± 84.43	0.374
Prothrombin time, INR	0.98 ± 0.02	0.96 ± 0.21	0.261	0.93 ± 0.10	0.94 ± 0.06	0.369
ALB (g/L)	4.03 ± 0.47	3.75 ± 0.57	**<0.001**	4.00 ± 0.41	3.83 ± 0.50	**<0.001**
CRP (mg/L)	8.13(5.32–12.69)	7.23(4.65–11.82)	0.081	4.43(2.65–7.39)	5.50(3.15–9.47)	0.165
FPG (mmol/L)	6.25 ± 1.96	6.31 ± 3.97	0.761	6.35 ± 1.93	6.31 ± 2.90	0.658
HbA1c (%)	9.40 ± 2.41	9.61 ± 2.91	0.118	9.00 ± 2.19	9.05 ± 2.55	0.795
Liver measurement						
LSM (kPa)	8.36 ± 3.50	7.63 ± 2.90	**<0.001**	4.67 ± 2.14	5.28 ± 1.25	**<0.001**
CAP (dB/m)	289.54 ± 81.74	276.98 ± 75.49	**0.013**	247.85 ± 71.56	257.29 ± 73.12	**<0.001**

Data are presented as mean ± SD, number (%), or median (interquartile range). BMI, body mass index; WHR, waist to hip ratio; SBP, systolic blood pressure; DBP, diastolic blood pressure; HT, hypertension; TC, total cholesterol; TG, triglyceride; HDL-c, high-density lipoprotein cholesterol; LDL-c, low-density lipoprotein cholesterol; ALT, alanine aminotransferase; AST, aspartate aminotransferase; GGT, γ-glutamyl transferase; Cr, creatinine; eGFR, estimated glomerular filtration rate; PLT, platelet; INR, international normalized ratio; ALB, albumin; CRP, C-reactive protein; FPG, fasting plasma glucose; HbA1c, glycosylated hemoglobin; LSM, liver stiffness measurement; CAP, controlled attenuation parameter. Bold values represents as *p* < 0.05.

Among 39 patients (30.2%) diagnosed with advanced liver fibrosis at baseline, a transition to the F0-1 stage was observed at readmission. In these cases, no significant changes were observed in body composition. Among those diagnosed with non-advanced liver fibrosis at baseline, 27.6% (72/261) progressed to the F2-3 stage. There was a significant increase in ΔBMI, ΔFMI and ΔTFMI, and a significant reduction in ΔM/F and ΔA/T levels compared to those maintained non-advanced liver fibrosis at readmission (Table 2).

**Table 2:** Body composition changes in incident F0-1/F2-3 subjects.

Body composition variables	Advanced liver fibrosis at baseline(*n* = 129)			Non-advanced liver fibrosis at baseline(*n* = 261)		
	Incident F0-1(*n* = 39)	Advanced liver fibrosis (*n* = 90)	*p*	Incident F2-3(*n* = 72)	Non-advanced liver fibrosis(*n* = 189)	*p*
ΔBMI (kg/m^2^)	0.31(−0.75–1.42)	0.67(−0.29–3.49)	0.092	0.46(−0.46–1.55)	−0.33(−1.31–0.62)	**0.006**
ΔFMI (kg/m^2^)	0.29(−0.36–1.16)	0.69(−0.19–1.83)	0.163	0.66(−0.20–1.53)	−0.07(−0.67–0.67)	**0.006**
ΔMMI (kg/m^2^)	0.14(−0.58–0.92)	0.08(−0.56–0.57)	0.674	−0.08(−0.67–0.55)	0.36(−0.41–0.84)	0.224
ΔM/F (%)	−0.10(−0.57–0.20)	−0.28(−0.65–0.11)	0.248	−0.09(−0.34–0.08)	0.12(−0.14–0.37)	**0.037**
ΔTFMI (kg/m^2^)	0.09(−0.27–0.69)	0.60(−0.19–1.41)	0.052	0.53(−0.12–1.25)	−0.02(−0.5–0.42)	**0.010**
ΔASMI (kg/m^2^)	0.21(−0.30–0.49)	−0.01(−0.27–0.26)	0.119	−0.04(−0.43–0.26)	0.03(−0.23–0.37)	0.229
ΔA/T (%)	−0.03(−0.33–0.14)	−0.23(−0.67–0.06)	0.102	−0.09(−0.24–0.05)	0.06(−0.13–0.26)	**0.022**

Data are presented as median (interquartile range). BMI, body mass index; FMI, fat mass index; MMI, muscle mass index; M/F, muscle/fat mass ratio; TFMI, trunk fat mass index; ASMI, appendicular skeletal muscle mass index; A/T, appendicular skeletal muscle mass/trunk fat mass ratio. Bold values represents as *p* < 0.05.

### 3.2 Correlation of weight change patterns with liver fibrosis outcomes

Univariate correlation analysis demonstrated that ΔBMI was positively correlated with ΔLSM among patients at baseline, regardless of liver fibrosis grade (*r* = 0.160 and 0.158, respectively, *p* < 0.01; Supplementary Table S2). To further explore the effect of different BMI trends on the outcome of liver fibrosis, we categorized readmitted patients into four groups according to weight change patterns and used binary regression analysis.

After adjusting for all covariates, the stable obese group with non-advanced liver fibrosis at baseline exhibited a significantly higher risk of incident F2-3 (OR = 3.464; 95% CI = 1.989–4.735). Conversely, among patients with advanced liver fibrosis at baseline, the stable obese group demonstrated the lowest risk of incident F0-1 (OR = 0.352; 95% CI = 0.137–0.562; Table 3).

**Table 3:** Association of weight changes with incident liver fibrosis risk among readmitted patients.

Incident F2-3 among non-advanced liver fibrosis at baseline	Weight change patterns			
	Stable non-obese (reference)	Weight loss	Weight gain	Stable obese
Events/total	25/110	23/88	12/24	12/39
unadjusted	1.000	0.812(0.504–1.238)	2.029(1.052–3.013)*	4.942(2.701–7.144)*
Model 1	1.000	0.860(0.317–1.376)	1.519(1.002–2.070)*	3.277(1.545–5.054)*
Model 2	1.000	0.870(0.168–1.313)	2.065(1.550–2.563)*	3.464(1.989–4.735)*
Incident F0-1 among advanced liver fibrosis at baseline				
Events/total	13/41	18/43	2/19	6/26
unadjusted	1.000	1.804(1.217–2.475)*	1.734(0.692–2.742)	0.560(0.236–0.960)*
Model 1	1.000	1.100(0.374–2.036)	0.942(0.814–1.989)	0.586(0.358–0.794)*
Model 2	1.000	0.609(0.166–1.106)	0.681(0.063–1.252)	0.352(0.157–0.562)*

Data are presented as OR (95% CI). Model 1: adjusted for age, gender; Model 2: adjusted for age, gender, obesity, hypertension, ΔSBP, ΔDBP, ΔWHR, ΔTG, ΔTC, ΔHDL-c, ΔLDL-c, ΔALT, ΔAST, ΔGGT, ΔALB, drug use. OR, odds ratio; CI, confidence interval; SBP, systolic blood pressure; DBP, diastolic blood pressure; TC, total cholesterol; TG, triglyceride; HDL-c, high-density lipoprotein cholesterol; LDL-c, low-density lipoprotein cholesterol; ALT, alanine aminotransferase; AST, aspartate aminotransferase; GGT, γ-glutamyl transferase; ALB, albumin. *p < 0.05.

### 3.3 Binary logistic regression analysis of body composition changes and liver fibrosis outcomes

Among individuals with advanced liver fibrosis at baseline, all body composition metrics were significantly correlated with ΔLSM except for ΔM/F (Supplementary Table S2). Although a positive association between ΔBMI and ΔLSM was identified, contrasting results were found for muscle and fat. Specifically, ΔFMI and ΔTFMI were positively correlated with ΔLSM, while ΔMMI, ΔASMI and ΔA/T were negatively correlated with ΔLSM. Furthermore, among the fat mass metrics, ΔTFMI exhibited the most powerful relationship with ΔLSM (*r* = 0.276, *p* = 0.004; Supplementary Table S2). Similarly, we performed binary regression analysis in order to further investigate the effect of body composition changes on liver fibrosis outcomes.

**Figure 2:**
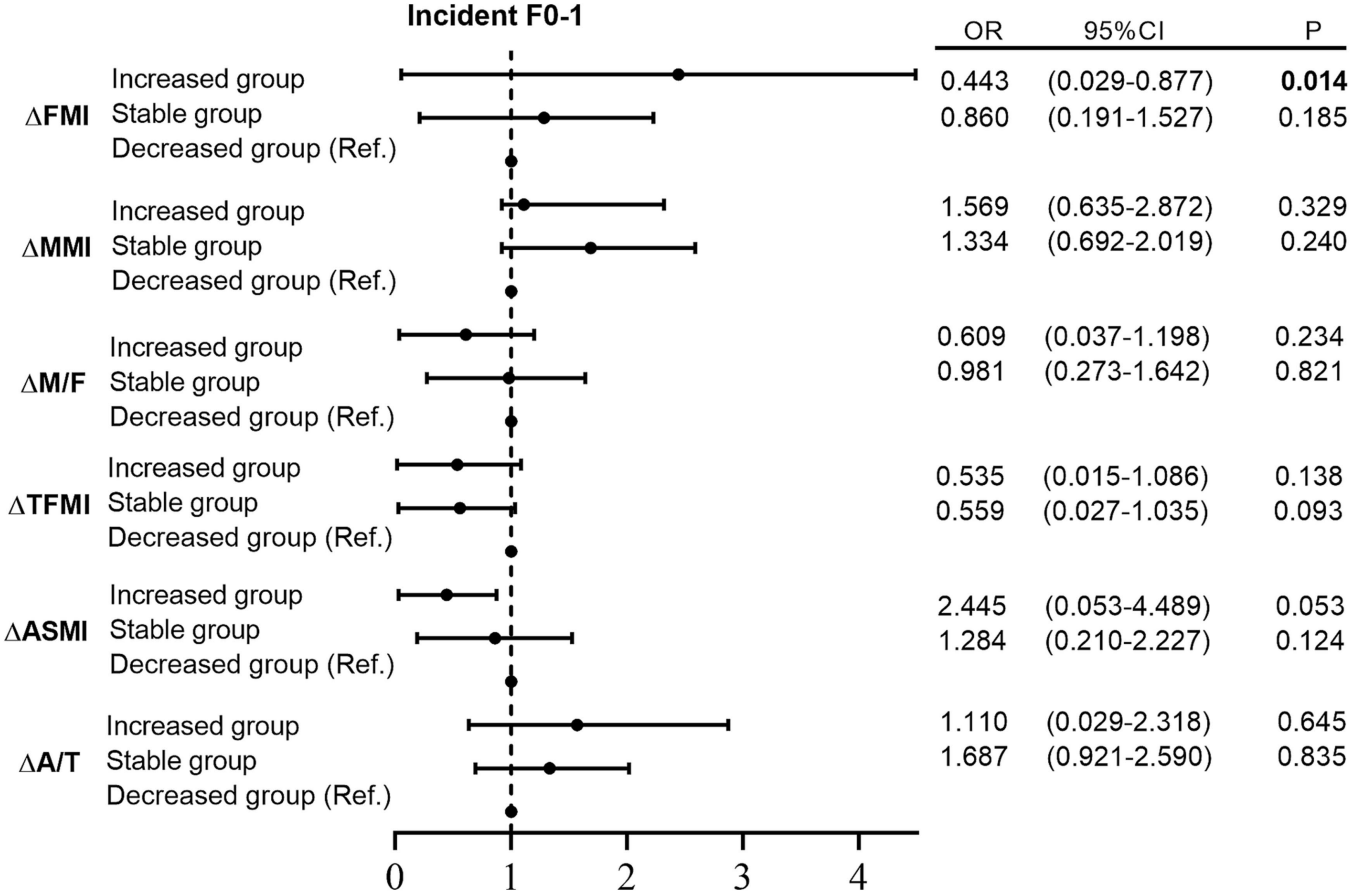
Binary logistic regression analysis between different trends of body composition and incident liver fibrosis Note: adjusted for age, gender, obesity, hypertension, drug use, ΔSBP, ΔDBP, ΔWHR, ΔTG, ΔTC, ΔHDL-c, ΔLDL-c, ΔALT, ΔAST, ΔGGT, and ΔALB. SBP, systolic blood pressure; DBP, diastolic blood pressure; TG, triglycerides; TC, total cholesterol; ALT, alanine aminotransferase; AST, aspartate aminotransferase; HDL-c, high-density lipoprotein cholesterol; LDL-c, low-density lipoprotein cholesterol; ALT, alanine aminotransferase; AST, aspartate aminotransferase; GGT, *γ*-glutamyl transferase; ALB, albumin; FMI, fat mass index; MMI, muscle mass index; M/F, muscle/fat mass ratio; TFMI, trunk fat mass index; ASMI, appendicular skeletal muscle mass index; A/T, appendicular skeletal muscle mass/trunk fat mass ratio.

After adjusting for confounders, the increased FMI group showed a significantly greater risk of incident F2-3 compared to the decreased group (*p* < 0.001; Figure 2). This trend was also observed in TFMI (*p* < 0.05). When FMI and TFMI increased, the risk of incident F2-3 was 3.618 and 3.601 times higher, respectively, in comparison to the decreased group (FMI: OR = 3.618, 95% CI = 1.794–5.739, *p* < 0.001; TFMI: OR = 3.601, 95% CI = 1.462–5.870, *p* = 0.002). Additionally, the increased MMI group appeared to have a slight advantage in preventing incident F2-3 compared to the stable group. This trend was also evident for the ASMI and A/T.

However, among individuals with non-advanced liver fibrosis at baseline, only ΔFMI was significantly correlated with ΔLSM (Supplementary Table S2). Moreover, the increased FMI group exhibited a significantly lower probability of incident F0-1 than the decreased group after adjusting for confounders (*p* = 0.014; Supplementary Figure S1).

## 4 Discussion

This study investigated the association between body composition changes and the outcome of liver fibrosis in a cohort of 390 patients with T2DM. Our findings indicated that middle-aged and elderly readmitted patients with T2DM who have incident advanced liver fibrosis tended to have higher BMI, FMI, and TFMI values, while M/F and A/T values were lower. Those who maintained stable obesity exhibited the highest risk of developing incident advanced liver fibrosis among non-advanced at baseline. Furthermore, subregional analysis demonstrated that non-advanced liver fibrosis patients at baseline with significant changes in FMI and TFMI were prone to develop incident advanced liver fibrosis. Conversely, Increased MMI, ASMI and A/T reduced the risk of developing incident advanced liver fibrosis. These findings highlight the potential of optimizing weight management strategies as a means of mitigating the risk of liver fibrosis in patients with T2DM.

Patients with T2DM who had advanced liver fibrosis at baseline exhibited more severe lipid metabolism disorders compared to non-advanced liver fibrosis adults, characterized by elevated TC and TG levels. Reaching the cirrhosis stage is uncommon for mild fibrosis (F1), which is generally recognized as an initial phase of NAFLD (27). However, in the context of obesity and T2DM, a considerable number of patients with fibrosis may exhibit heightened susceptibility to accelerated disease progression toward more severe liver pathology (28, 29). In addition to obesity status, there is growing research interest in investigating correlation between weight fluctuations and their impact on health outcomes, given the prevalent occurrence of weight changes throughout adulthood (30, 31). The finding of a large prospective cohort study revealed that both obesity and weight gain were positively associated with liver fibrosis progression (17). Our findings consistently highlight that stable obese individuals with T2DM face the highest risk of incident advanced liver fibrosis, underscoring the vulnerability of obese and diabetic patients and emphasizing the need for more vigilant screening measures. Elevated intrahepatic triglycerides resulting from excessive delivery of FFAs to the liver and musculoskeletal tissue contribute to fat accumulation in the liver, promoting hepatic fibrotic lesions (32). Hence, controlling weight gain emerges as a crucial strategy for reducing the risk of liver fibrosis.

Sarcopenia and NAFLD often coexist and may worsen chronic inflammation and oxidative stress linked to obesity (33). The novel results of our study demonstrated that increased ASMI and A/T over time were beneficial for preventing the progression of advanced liver fibrosis, regardless of baseline ASMI and A/T. Skeletal muscle is acknowledged as an endocrine organ to release various myokines including irisin and interleukin-6 (34, 35). Exercise is known to stimulate the release of healthy myokines and promote muscle hypertrophy. Irisin, stimulated by exercise, activates peroxisome proliferator activated receptor *α* signaling and is pivotal in fatty acid *β*-oxidation in the liver, resulting in improvements in hepatic steatosis and insulin sensitivity accompanied by the upregulation of fibroblast growth factor 21 (36, 37). Therefore, skeletal muscle could potentially influence the development or amelioration of liver fibrosis by releasing favorable myokines. Furthermore, insufficient muscle mass leads to physical disability which reduces energy expenditures, increases the risk of obesity, and contributes to hepatic steatosis (15). When categorized based on A/T trends, the findings revealed that individuals with a decreased muscle/fat ratio exhibited increased susceptibility to incident advanced liver fibrosis, even if they were non-advanced at baseline.

Chronic inflammation could serve as a crucial connection between decreased muscle mass and liver fibrosis (38). Growth differentiation factor (GDF-15), an inflammatory and sarcopenic biomarkers, was found to be associated with hepatitis and liver fibrosis in NAFLD (39). Consequently, elevated GDF-15 level may potentially influence the development of sarcopenia and the occurrence of advanced liver fibrosis. Moreover, lower serum vitamin D levels may lead to decreased muscle mass and incident advanced liver fibrosis because vitamin D deficiency is correlated to both sarcopenia and NAFLD (40).

Reduced muscle mass and increased adiposity are significant independent contributors for the pathogenesis of diabetes. Investigations have demonstrated that each one SD increase in BMI among Asians is associated with a 1.52–1.59 times higher likelihood of developing diabetes (41). However, the progression of liver fibrosis varies among individuals due to multiple factors. We observed that patients who experienced weight gain or remained stable obese group exhibited a significantly higher risk of incident advanced liver fibrosis compared to the stable non-obese group. Additionally, the increased MMI group appeared to slightly more favorable in preventing advanced liver fibrosis when compared with the stable group. Similar trends were observed in ASMI and A/T, suggesting that changes in BMI alone may not accurately reflect changes in liver fibrosis among patients with T2DM.

The main strength of this study lies in its design as a cohort study with a substantial number of participants. We also excluded individuals with irregular thyroid function and chronic kidney diseases, which were linked to the advancement of NAFLD or sarcopenia (42, 43). Furthermore, data on the correlation between changes in body composition and incident advanced liver fibrosis at baseline and readmission is a novel contribution to the filed. However, there are several limitations needed to consider. Firstly, despite being a retrospective study, the relatively short follow-up period in our study may have limited the ability to thoroughly assess the relationship between long-term changes in body composition and the outcome of advanced liver fibrosis. Extending the follow-up time could offer more robust insights into these relationships. And the findings are associative and not causal. Secondly, the study population primarily consisted of middle-aged and elderly individuals from a single center. This may restrict the applicability of the results to other age groups. And missing data may bias the results. Thirdly, this study lacks mechanistic insight. However, following publications describing novel interactions between liver fibrosis and energy metabolism through experimental studies and transgenic models, it becomes imperative to validate these hypotheses in relevant human populations *in vivo*. Fourthly, we did not utilize other non-invasive markers like Fibrosis 4 score (FIB-4) for liver fibrosis diagnosis due to the limited number of liver fibrosis events observed. The use of such an index might resulted in overlooking many liver fibrosis events. Instead, we diagnosed incident advanced liver fibrosis using LSM rather than liver biopsy. Although liver biopsy serves as the gold standard (44, 45), conducting invasive test in a large population-based investigation was impractical. Furthermore, while we made adjustments for known confounders in our analyses, there may be unmeasured variables that could still influence the observed associations. We recommend that future research should aim to include a more comprehensive assessment of these confounders and consider longitudinal data to better capture the dynamic relationships between these factors.

## 5 Conclusion

We observed that persistent obesity and weight accumulation were associated with an elevated hazard of incident advanced liver fibrosis in adults with T2DM. Additionally, an increased TFMI may promotes the progression of liver fibrosis, while maintaining a balanced muscle/fat ratio could contribute to preventing advanced liver fibrosis progression.

## Data Availability

The original contributions presented in the study are included in the article/Supplementary material, further inquiries can be directed to the corresponding author.

## Supplementary material

SUPPLEMENTARY FIGURE S1 Binary logistic regression analysis between different trends of body composition and incident F0-1.

## Glossary

T2DM: type 2 diabetes mellitus
BMI: body mass index
FMI: fat mass index
MMI: muscle mass index
TFMI: trunk fat mass index
ASMI: appendicular skeletal muscle mass index
M/F: body muscle mass / body fat mass
A/T: appendicular skeletal muscle mass /trunk fat mass
TE: transient elastography
LSM: liver stiffness measurement
CAP: controlled attenuation parameter
IQR: interquartile range
NAFLD: nonalcoholic fatty liver disease
WHR: waist to hip ratio
TC: total cholesterol
TG: triglyceride
HDL-c: high-density lipoprotein cholesterol
LDL-c: low-density lipoprotein cholesterol
ALT: alanine aminotransferase
AST: aspartate aminotransferase
GGT: *γ*-glutamyl transferase
Cr: creatinine
CRP: C-reactive protein
PLT: platelet
INR: international normalized ratio
ALB: albumin
FPG: fasting plasma glucose
eGFR: estimated glomerular filtration rate
HbA1c: glycosylated hemoglobin
SBP: systolic blood pressure
DBP: diastolic blood pressure
DEXA: dual-energy X-ray absorptiometry
SDs: standard deviations
ORs: odds ratios
CIs: confidence intervals
GDF-15: growth differentiation factor
FIB-4: fibrosis 4 score
