# Supplementary material for: Association between changes in body composition and progression of liver fibrosis in patients with type 2 diabetes mellitus
Source: Front Nutr. 2024 Oct 21;11:1476467. doi: 10.3389/fnut.2024.1476467 (PMC11532110; doi:10.3389/fnut.2024.1476467)
Supplement: Supplementary file 1 [file Table_1.docx]

**Table S1.** Comparison of body composition between baseline and readmitted subjects

| **Body composition measurement** | **Advanced liver fibrosis**  **at baseline**  **（n=129）** | **Readmitted patients**  **（n=129）** | **P** | **Non-advanced liver fibrosis**  **at baseline**  **（n=261）** | **Readmitted patients**  **（n=261）** | **P** |
| --- | --- | --- | --- | --- | --- | --- |
| FMI (kg/m^2^) | 7.75(6.40-9.35) | 6.45(4.77-7.95) | **0.011** | 5.76(4.26-7.38) | 7.71(6.37-9.53) | 0.768 |
| MMI (kg/m^2^) | 15.77(14.52-17.29) | 16.07(14.76-17.24) | 0.258 | 16.90(15.47-17.95) | 16.79(15.57-18.23) | 0.423 |
| M/F (%) | 2.17(1.74-2.75) | 2.44(1.93-3.35) | **0.026** | 2.69(2.11-3.83) | 2.11(1.66-2.72) | 0.619 |
| TFMI (kg/m^2^) | 4.73(3.84-5.67) | 3.69(2.64-4.67) | **0.028** | 3.35(2.49-4.37) | 4.78(3.93-5.79) | 0.526 |
| ASMI (kg/m^2^) | 6.39(5.70-7.23) | 6.52(5.84-7.30) | 0.402 | 7.01(6.24-7.78) | 7.00(6.30-7.80) | 0.823 |
| A/T (%) | 1.52(1.16-1.89) | 1.83(1.32-2.47) | **0.050** | 1.96(1.49-2.66) | 1.43(1.14-1.91) | 0.365 |

Data are presented as median (interquartile range).

FMI, fat mass index; MMI, muscle mass index; M/F, muscle/fat mass ratio; TFMI, trunk fat mass index; ASMI, appendicular skeletal muscle mass index; A/T, appendicular skeletal muscle mass/trunk fat mass ratio.
