# Supplementary material for: Association between changes in body composition and progression of liver fibrosis in patients with type 2 diabetes mellitus
Source: Front Nutr. 2024 Oct 21;11:1476467. doi: 10.3389/fnut.2024.1476467 (PMC11532110; doi:10.3389/fnut.2024.1476467)
Supplement: Supplementary file 2 [file Table_2.docx]

**Table S2** Pearson’s correlation between body composition changes and liver stiffness among readmitted T2DM patients

|  | | Δ**LSM** | |
| --- | --- | --- | --- |
|  | | Advanced liver fibrosis  at baseline | Non-advanced liver fibrosis  at baseline |
| Δ**BMI** | *r* | **0.160** | **0.158** |
|  | *p* | **0.006** | **0.025** |
| Δ**FMI** | *r* | **0.130** | **0.185** |
|  | *p* | **0.029** | **0.010** |
| Δ**MMI** | *r* | **-0.121** | -0.076 |
|  | *p* | **0.042** | 0.293 |
| Δ**M/F** | *r* | -0.102 | -0.096 |
|  | *p* | 0.086 | 0.186 |
| Δ**TFMI** | *r* | **0.276** | 0.027 |
|  | *p* | **0.004** | 0.712 |
| Δ**ASMI** | *r* | **-0.255** | -0.030 |
|  | *p* | **<0.001** | 0.674 |
| Δ**A/T** | *r* | **-0.059** | -0.118 |
|  | *p* | **0.021** | 0.105 |

T2DM, type 2 diabetes mellitus; BMI, body mass index; FMI, fat mass index; MMI, muscle mass index; M/F, muscle/fat mass ratio; TFMI, trunk fat mass index; ASMI, appendicular skeletal muscle mass index; A/T, appendicular skeletal muscle mass/trunk fat mass ratio; LSM, liver stiffness measurement.
