# Supplementary figures and images for: Association between changes in body composition and progression of liver fibrosis in patients with type 2 diabetes mellitus
Source: Front Nutr. 2024 Oct 21;11:1476467. doi: 10.3389/fnut.2024.1476467 (PMC11532110; doi:10.3389/fnut.2024.1476467)

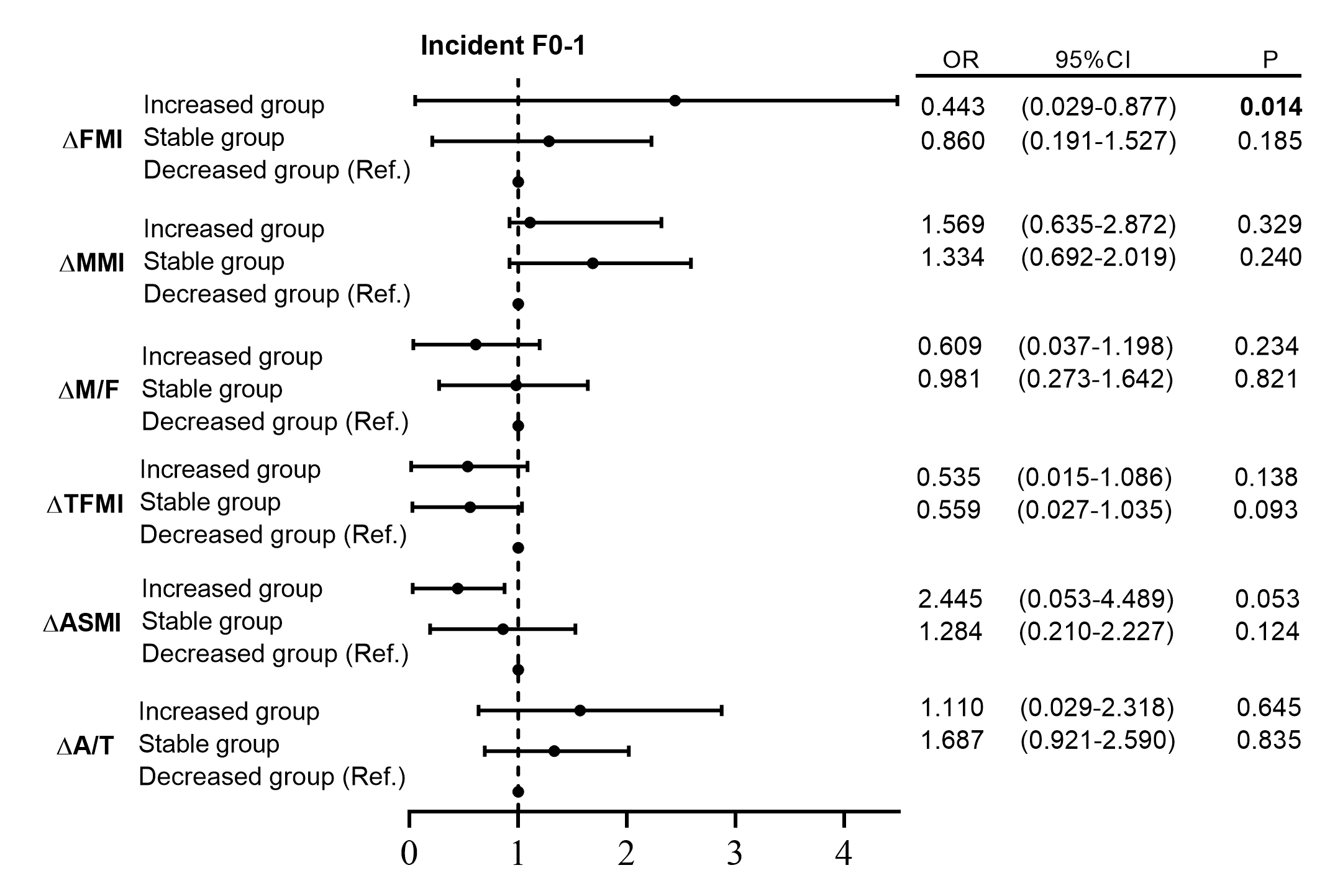

Supplement: SUPPLEMENTARY FIGURE S1 — Binary logistic regression analysis between different trends of body composition and incident F0-1. [file Image_1.tif]
